# The efficacy and safety of nine South African medicinal plants in controlling *Bacillus anthracis Sterne* vaccine strain

**DOI:** 10.1186/s12906-015-0980-1

**Published:** 2016-01-08

**Authors:** Ishaku Leo Elisha, Jean-Paul Dzoyem, Francien S. Botha, Jacobus Nicolaas Eloff

**Affiliations:** 1 Phytomedicine Programme, Department of Paraclinical Sciences, Faculty of Veterinary Science, University of Pretoria, Private Bag X04, Onderstepoort, 0110 Pretoria, South Africa; 2 Present address: Drug Development Section, Biochemistry Division, National Veterinary Research Institute, P.M.B. 01, Vom, Plateau State Nigeria; 3 Present address: Department of Biochemistry, Faculty of Science, University of Dschang, P.O. Box 67, Dschang, Cameroon

**Keywords:** *Bacillus anthracis*, Medicinal plants, Antibacterial activity, Total activity, Cytotoxicity, Selectivity index

## Abstract

**Background:**

Anthrax is a zoonotic disease caused by *Bacillus anthracis*, a Gram-positive spore-forming bacterium. The presence of the bacteria and the toxins in the blood of infected hosts trigger a cascade of pathological events leading to death. Nine medicinal plants with good activities against other bacteria were selected to determine their *in vitro* antibacterial activity against *Bacillus anthracis* Sterne strain*.* The cytotoxicity of the extracts on Vero kidney cells was also determined.

**Results:**

The minimum inhibitory concentration (MIC) values of the extracts against *Bacillus anthracis* Sterne strain ranged from 0.02 to 0.31 mg/ml. Excellent MIC values were observed for the following plant species: *Maesa lanceolata* (0.02 mg/ml), *Bolusanthus speciosus*, *Hypericum roeperianum*, *Morus mesozygia* (0.04 mg/ml) and *Pittosporum viridiflorum* (0.08 mg/ml). The total antibacterial activity of the extracts ranged from 92 to 5562 ml/g. Total activity presents the volume to which the extract from 1 g of plant material can be diluted and still inhibit microbial growth. *Maesa lanceolata* and *Hypericum roeperianum* had the highest total activity with values of 5562 and 2999 ml/g respectively. The extracts of *Calpurnia aurea* had the lowest total activity (92 ml/g). The cytotoxicity determined on Vero cells indicated that most of the extracts were relatively non-toxic compared to doxorubicin (LC_50_ 8.3 ± 1.76 μg/ml), except for the extracts of *Maesa lanceolata*, *Elaeodendron croceum* and *Calpurnia aurea* with LC_50_ values at 2.38 ± 0.25, 5.20 ± 0.24 and 13 ± 2.26 μg/ml respectively. The selectivity index (SI) ranged from 0.02 to 1.66. *Hypericum roeperianum* had the best selectivity index, (SI = 1.66) and *Elaeodendron croceum* had lowest value (SI = 0.02).

**Conclusions:**

The crude acetone extracts of the selected plant species had promising antibacterial activity against *Bacillus anthracis. Maesa lanceolata* extracts could be useful as a disinfectant and *Hypericum roeperianum* could be useful to protect animals based on its high total activity and selectivity index. Further investigation of these plant extracts may lead to the development of new therapeutic agents to protect humans or animals against anthrax.

## Background

Anthrax is a serious zoonotic disease of great economic and social importance [1]. It is caused by *Bacillus anthracis*, a Gram-positive spore-forming bacterium [2]. The disease can affect most mammals and several species of birds, but is particularly important in herbivores, such as cattle, sheep and goats [1, 2]. Humans contract anthrax by handling infected animals or products of animals that have died from the disease [3]. Once infection sets in, spores germinate into capsulated bacilli capable of producing toxins. The presence of the bacteria and their toxins in the blood of the infected host triggers a cascade of pathological events leading to death [3]. *B. anthracis* has always been high on the list of agents that could be used in biological warfare and bioterrorism [4]. The disease is still endemic in many countries of Africa and Asia [3].

The major virulence factors of *B.anthracis* are encoded on two virulence plasmids pXO1 and pXO2 [5]. The tri-toxin bearing plasmid pXO1 codes for three toxins, which cause haemorrhage, oedema, and necrosis [6]. They comprise the lethal factor, oedema factor, and the protective antigen, in the host cell receptor component. The exotoxins are binary, with the protective antigen acting as the binding domain that allows entry of the toxin into the host cell. The smaller capsule bearing plasmid pXO2 encodes three genes (cap B, cap C, and cap A) involved in the synthesis of the poly-D- glutamyl capsule that inhibits host phagocytosis of the vegetative form of *B. anthracis*. Both plasmids are necessary for full virulence; loss of either results in an attenuated strain. Sterne *B. anthracis* strain, carries pXO1 and therefore can synthesise exotoxin, but does not have a capsule [5]. Microbiology laboratories use *B. anthracis* Sterne strain for accurate identification and diagnosis of anthrax, and occasionally for anthrax research [7].

*B. anthracis* spores have the capacity to contaminate a given area for a long time, because of their intrinsic ability to survive different environmental conditions and chemical disinfectants [2, 8]. Anthrax spores have been isolated after 60 years from contaminated sites [8, 9]. *B. anthracis* has been recovered from animal bones estimated to be over 200 years old in the Kruger National Park in South Africa [10].

Control measures employed during anthrax outbreaks include vaccination of livestock using the avirulent *B. anthracis* Sterne strain [7], burial or burning of dead animals and calcium oxide (lime) application on burial sites [2, 11]. Anthrax infections can be treated effectively with antibiotics, provided treatment is started early. Penicillin has been the antibiotic of choice for many years [1]. Where the use of penicillin is contraindicated or ineffective, ciprofloxacin and doxycycline are good alternatives [4]. *B. anthracis* natural or acquired resistance to broad spectrum antibiotics [12–14], as well as poor penetration of doxycycline into the central nervous system of infected individuals, necessitate the search for new antimicrobials that could offer effective and potent alternatives in face of bioterrorist attack or anthrax epidemics [4].

Plants have always played a central part in combating ailments in humans and livestock in many indigenous communities [15]. Herbal extracts or decoctions from different plant parts have been used in both ethnoveterinary and ethnomedicinal practices to treat anthrax in animals and humans [16–19]. Traditional use of the following plants have been documented in the treatment of anthrax in livestock and humans, *Senna italica*, *Teucrium africanum*, *Ptaeroxylon obliquum*, *Achyrospermum schimperi*, *Teucrium polium* [16, 17, 20].

The Phytomedicine Programme, University of Pretoria, has screened over 700 acetone leaf extracts of more than 530 tree species for their antimicrobial properties beneficial to man and animals [21]. Nine plant species with high antibacterial activities were selected from the database and tested against *Bacillus anthracis* Sterne strain. The cytotoxicity was also determined using the MTT assay against Vero kidney cells.

## Methods

### Collection of plant material

The leaves of *Heteromorpha arborescens* (Spreng.) Chan. & Schltdl, *Bolusanthus speciosus* (H. Bolus) Harms, *Maesa lanceolata* Forssk, *Elaeodendron croceum* (Thunb.) DC, *Pittosporum viridiflorum* Sims, *Hypericum roeperianum* G.W. Schimp.ex A.Rich. Var. roeperianum, *Morus mesozygia* Stapf ex A.Chev., *Cremaspora triflora* (Thonn.) K.Schum. and *Calpurnia aurea* (Aiton) Benth ssp *aurea* were collected in the summer of 2013, from the University of Pretoria Botanical Garden, Pretoria National Botanical Garden and Lowveld National Botanical Garden in Nelspruit, Mpumalanga Province South Africa. Voucher specimens were prepared and deposited in the HGWJ Schweickerdt Herbarium of the University of Pretoria (PRU) (Table 1).

**Table 1:** A summary of the nine medicinal plants investigated for activity against *Bacillus anthracis* Sterne strain

Plant name	Family	Common name(s)	Parts used	Traditional uses	Voucher No.
*Hypericum roeperianum*	Hypericaceae	Large-leaved Curry Bush	Leaves	Infections [42]	PRU 120126
*Cremaspora triflora*.	Rubiaceae	Synonym: Cremaspora coffeoides Hemsl. (1896).	Bark, roots	Tooth ache, rheumatism, swellings, intestinal parasites diuretic	PRU 120129
*Heteromorpha arborescens*	Apiaceae	Parsley tree (Eng.); Wildepietersielie (Afr.)	Leaves, roots	Abdominal pains, intestinal worms, nervous and mental disorders, shortness of breath, coughs, dysentery, headaches, dysmenorrhoea, aphrodisiac, gall sickness and red water in livestock [18, 43]	PRU 120026
*Pittosporum viridiflorum*	Pittosporaceae	Cheesewood (E), Kasuur (A), umVusamvu (Z)	leaves, bark	Increase lactation in cows, camels, goats and sheep, tonic, infectious diseases, inflammations and chewing stick (oral hygiene), oral fungal infections in HIV positive patients [18, 44]	PRU 120025
*Bolusanthus speciosus*	Fabaceae	Tree wisteria (English); Vanwykshout (Afrikaans); Mogaba (Northern Sotho); Motsokophala (Tswana); Mukambana (Venda), umHolo (Zulu)	bark	Abdominal cramps, vomiting, tuberculosis, venereal diseases, anti-termite [45]	PRU 120027
*Calpurnia aurea*	Fabaceae	Common Calpurnia, Calpurnia, Wild Geelkeurboom, Geelkeur (Afr.)	leaves, roots, seeds	Malaria, wound healing, stomach ache, headaches, eye infections, rheumatism, diarrhoea, leishmaniasis, taeniasis, trachoma, allergic rashes caused by caterpillar, elephantiasis, swellings, fungal skin diseases, excessive menstrual flow, amoebiasis, syphilis, giardiasis, rabies, diabetes, abscess, hypertension, maggots in wounds, control of ticks, lice and mites in chickens, mastitis in cows, other gastrointestinal tract infections in livestock [38, 46]	PRU 120024
*Maesa lanceolata*	Maesaceae	False assegai ( Eng. ); Valsassegaai (Afr.)	Leaves, roots, fruits	Malaria, dysentery, diarrhoea, dermatosis, hypertension, sore throat, hepatitis, cholera, taeniasis, rheumatic arthritis, pimples, flu, syphilis, gonorrhoea, stomach ache, appetizer [33, 47].	PRU 120125
*Elaeodendron croceum*	Celastraceae	Saffron; saffron wood, forest saffron (Eng.); saffraan, bossafraan (Afr.)	Leaves, bark, roots	Cleaning of the digestive tract, chest congestion [34, 35].	PRU 120127
*Morus mesozygia*	Moraceae	Black mulberry or African mulberry	Leaves, bark, roots	Arthritis, debility, malnutrition, gastroenteritis, rheumatism, venereal diseases, wound infections, dermatitis, syphilis, malaria, fever, pain, depression, peptic ulcers [48]	PRU 120128

### Preparation of extracts

The finely ground dry leaf powder (3.0 g) of each plant was extracted with 30 ml acetone (technical quality; Merck chemicals (Pty) Ltd Wadeville, South Africa). Acetone is the most effective extractant for antimicrobial compounds from plants based on several parameters [22]. The suspension was shaken vigorously in 50 ml polyester centrifuge tubes and centrifuged at 4000 × g for 10 minutes (Hettich Centrifuge, Rotofix 32A, Labotec, Johannesburg, South Africa). The supernatants were decanted into preweighed glass vials after filtering with Whatman No. 1 filter paper and concentrated to dryness under a stream of cold air. The dried extracts were made up to a concentration of 10 mg/ml (stock solution) in acetone to be used in subsequent assays and stored at 5 °C in tightly stoppered glass tubes.

### Test organism

*Bacillus anthracis* Sterne strain cultures used to determine minimum inhibitory concentration (MIC) were obtained from the Department of Veterinary and Tropical Diseases, Faculty of Veterinary Science, University of Pretoria. The bacterial cultures were maintained on Müller-Hinton agar (Merck, South Africa) at 4 °C and incubated in Müller- Hinton broth at 37 °C prior to the determination of MICs.

### Antibacterial screening

The microplate serial dilution method of Eloff [23] using *p-* iodonitrotetrazolium violet as growth indicator was used to determine the antibacterial activity of the extracts. This method allows the calculation of minimal inhibitory concentration (MIC) values for active plant extracts against microbes. The experiment was performed in triplicate and repeated thrice to confirm activities.

Freshly inoculated bacterial culture of *Bacillus anthracis* Sterne strain in Müller Hinton broth were incubated overnight at 37 °C. The culture was adjusted to a McFarlane standard 1 which is equivalent to 3 × 10^8^ colony forming units/ml. to use as inoculum, A two-fold serial dilution of plant extract (100 μl) was prepared in 96-well microtitre plates, and 100 μl of the inoculum were added to each well providing a 50 % inoculum. The presence of bacterial growth was detected by adding to each well 40 μl of 0.2 mg/ml INT (ρ-iodonotrotetrazolium violet, Sigma South Africa). Microplates were examined after 30-120 minutes incubation. Bacterial growth was indicated by a red colour change caused when INT is reduced to formazan. The lowest concentration at which a decrease in the red colour is apparent compared to the next dilution was taken as the MIC value. Gentamicin was included as positive standard in the assay, and acetone was included as a solvent control. To compare the activity of different plant extracts, the total activity in ml/g was calculated by dividing the total mass in mg extracted from 1 g of dried plant material by the MIC in mg/ml [24]. This provides the volume to which the extract from 1 g of plant material can be diluted and still inhibited the growth of the microbe. To isolate new promising compounds from plants the MIC is important, but to consider the use of an extract not only the MIC but also the quantity extracted by the solvent i.e. total activity is important.

### Cytotoxic activity

The cytotoxicity of the acetone extracts against Vero monkey kidney cells was determined by using the 3-(4,5-dimethylthiazol-2-yl)-2, 5-diphenyltetrazolium bromide (MTT) reduction assay as previously described by Mosmann [25] with slight modifications. Cells were seeded at a density of 1 × 10^5^ cells/ml (100 μl) in 96-well microtitre plates and incubated at 37 °C and 5 % CO_2_ in a humidified environment. After 24 hours incubation, 100 μl each of differing extract concentrations were added to the wells containing cells. Doxorubicin was used as a positive control. A suitable blank control with equivalent concentrations of acetone was also included and the plates were incubated for 48 h in a CO_2_ incubator. Thereafter, the medium in each well was aspirated from the cells, cells were washed with PBS, and finally 200 μl fresh medium was added to each well. Thirty microlitre of MTT (5 mg/ml in PBS) was added to each well and the plates were incubated at 37 °C for 4 h. The medium was aspirated from the wells and DMSO was added to solubilise the formed formazan crystals. The absorbance was measured using a BioTek Synergy microplate reader at 570 nm. The percentage of cell growth inhibition was calculated based on a comparison with untreated cells. The selectivity index values were calculated by dividing cytotoxicity LC_50_ values by the MIC values in the same units (mg/ml). The result provides an indication of the safety to toxicity ratio.

### Statistical analysis

Experimental data were analysed using Microsoft Excel Version 2010. The mean values were calculated and reported as the mean ± standard deviation (SD).

## Results and discussion

### Extract yield and total antibacterial activity

Three reasons have been outlined as the basis for screening medicinal plants: (1) finding new phytocompounds for possible drug development (2), verifying claims of both ethnomedicinal and ethnoveterinary applications by traditional users, and (3) to develop phytomedicines for use as herbal medicines [26]. Even if results obtained from extract evaluation are expressed in quantitative terms, it is often difficult to compare different plants with the outcomes obtained [26]. The calculation of total activity (TA) of plant extracts in antimicrobial assays provide additional confirmation of their potential use. Total activity provides the volume to which active constituents in plant material can be diluted and still inhibit the growth of the tested microorganism, expressed in millilitre per gram [24]. Combined with bioautography it also indicates the quantity and quality of active constituents present in the plant extract [27]. The total antibacterial activity of the acetone leaf extracts ranged from 92 to a surprising 5562 ml/g. *Maesa lanceolata* and *Hypericum roeperianum* had the highest total activity, with values at 5562 and 2999 ml/g respectively. The high activities observed resulted from excellent MIC values, 0.02 and 0.04 mg/ml and good extraction yields at approximately 12 and 11.12 % respectively (Table 2). The low MIC value of an extract may not always mean that it is the best extract or the best fraction for further investigation. Although, all the plant extracts tested against *B. anthracis* had promising activity (Table 2), their low extract yield affected the total activity values when compared to *Maesa lanceolata* and *Hypericum roeperianum*. For example, *Morus mesozygia*, *Bolusanthus speciosus*, and *Pittosporum viridiflorum* had a lower total activity due to low extraction yield.The very low total activity of *Calpurnia aurea* can be ascribed to the high MIC value and low percentage extract yield. The higher the total activity, the greater is the potential for application of the specific plant extract [24]. The concept of total activity is also useful when isolating bioactive compounds to determine if there is a loss of activity and if synergism may be involved [26].

**Table 2:** The quantity extracted from 1 gram of dry plant material, MIC values, total activity, cytotoxicity and selectivity index (SI)

Plant names	Quantity extracted per 1 gram (mg/g)	Mean MIC (mg/ml)	Total Activity (ml/g)	Cytotoxicity (μg/ml)	SI
*Hypericum roeperianum*	119.97	0.04	2999	66.2 ± 0.02	1.66
*Cremaspora triflora*	20.17	0.16	126	57.4 ± 2.94	0.36
*Heteromorpha arborescens*	26.03	0.16	163	81.0 ± 7.6	0.51
*Pittosporum viridiflorum*	27.17	0.08	340	54.6 ± 14.3	0.68
*Bolusanthus speciosus*	23.03	0.04	576	52.8 ± 3.92	1.32
*Calpurnia aurea*	28.63	0.31	92	13.6 ± 2.26	0.04
*Maesa lanceolata*	111.23	0.02	5562	2.38 ± 0.25	0.12
*Elaeodendron croceum*	89.97	0.31	290	5.2 ± 0.24	0.02
*Morus mesozygia*	18.47	0.04	462	40.7 ± 1.54	1.02
Gentamicin	ND	0.0002	NA	NA	ND
Doxorubicin	NA	NA	NA	8.3 ± 1.76	ND

*NA* Not applicable, *MIC* Minimum inhibitory concentration. The standard deviation for the MICs (9 determinations) were 0.00

### Minimum inhibitory concentration

The mean MIC values of the extracts ranged from 0.02 to 0.31 mg/ml (Table 2). Amongst the selected extracts, *Pittosporum viridiflorum*, *Bolusanthus speciosus*, *Hypericum roeperianum*, *Morus mesozygia* and *Maesa lanceolata* had good activity (Table 2). Pauw and Eloff [21] judged that only MIC values ≤ 0.1 mg/ml should be considered as good antimicrobial activity. Nonetheless, the same authors while screening 714 acetone leaf extracts of 537 different tree species included MIC values ≤ 0.16 mg/ml as relevant because they determined activity at 0.08 and 0.16 mg/ml and not at 0.1 mg/ml. This inclusions was made in order to facilitate their statistical analyses, implying that such extracts also have the potential for further investigation. Consequently, *Cremaspora triflora* and *Heteromorpha arborescens* (MIC values 0.16 mg/ml) had potentially interesting activities against *B. anthracis*. The traditional uses of the plants are shown in Table 1.

There are reports of other crude plant extracts active against standard *B. anthracis* strains and their minimum inhibitory concentrations (MIC) values were determined. MICs were also determined for ethanolic extracts of the aerial parts of *Cissampelos mucronata* (0.195 mg/ml), aqueous root extracts of *Tephrosia villosa* (0.781 mg/ml) [28], aqueous extracts of *Combretum adenogonium* leaves(0.31 mg/ml) and the stem bark and root (2.5 mg/ml) [29]. Minimum bactericidal concentration (MBC) of *Teucrium polium* against *B. anthracis* is 10 mg/ml [20]. With the exception of *Elaeodendron croceum* and *Calpurnia aurea* all the results found in this study were between ten and 125 times higher than values previously reported. This may be due to the extractant, plant or bioassay that was used [22].

### Cytotoxicity

Despite a common belief that plant extracts and phytoconstituent are safe, many plant metabolites are very toxic [30]. *In vitro* toxicity screening is a compulsory aspect of the pilot safety assessment of plant extracts and compounds before further development and commercialisation [31]. The active ingredients of crude extracts are chemicals that may be similar to those in purified medications, and may have the same potential to cause serious adverse effects [30]. The *in vitro* cytotoxicity of the crude acetone leaf extracts of the nine selected plants are presented in Table 2. The extracts had varying degrees of toxicity on the Vero cell lines, with LC_50_ values ranging from 2.38 ± 0.25 to 81.0 ± 7.6 μg/ml. In classifying the safety of plant extracts, LC_50_ values > 20 μg/ml were considered non-toxic [32]. Hence, *Calpurnia aurea*, *Elaeodendron croceum* and *Maesa lanceolata* had a high cytotoxicity in this study. Previous studies on these plant species revealed their toxicities to different cell lines [33–36]. Cytotoxicity of natural and semi-synthetic analogs of alkylated benzoquinones isolated from the leaves and fruits of *Maesa lanceolata* against Human leukaemia (HL-60) cell lines have been reported, with LC_50_ ranging from 0.23-4.5 μg/ml [36]. The stem barks of *Calpurnia aurea* have previously been reported as poisonous to fish [37]. Similarly, Zorloni et al. [38] reported that both acetone and aqueous leaf extracts of *Calpurnia aurea* on ticks either immobilised or killed the arachnids. Our results agree with those of Yelani et al. [35], regarding the cytotoxicity of *Elaeodendron croceum* on Vero cells. They isolated five toxic triterpenoids 20-hydroxy-20-epi-tingenone, tingenone, tingenine B, 11-hydroxy-amyrin and naringenin from *Elaeodendron croceum* leaves.

*Hypericum roeperianum* had the highest selectivity index of 1.66. The higher the value of the selectivity index the safer the extract is (Table 2). This value shows the ratio of efficacy to toxicity helps to eliminate activity that may possibly be due to a general metabolic toxin [39]. The selectivity index differentiates between activity that is caused by general toxicity and one that is selectively toxic to the microorganism [39]. Although *Maesa lanceolata* had excellent MIC value and total antibacterial activity against *B. anthracis*, it was not suitable for further investigation because of its toxicity and low selectivity index (Table 2).

## Conclusions

In a review of anthrax in animals, Beyer and Turnbull [3] recounted different incidences where millions of livestock died from anthrax in Russia, Iran and South Africa. Regular infections of livestock with *B. anthracis* present a consequent risk to public health [8]. Sporadic outbreaks of anthrax occur frequently in Africa [1, 11], due to constraints of treatment, prevention and control measures. The expense of decontaminating polluted anthrax sites, and unavailability of approved disinfectants is high [2, 11]. In the face of these challenges, indigenous medicinal plant extracts may serve as potential alternatives to chemicals as disinfectants or decontaminants, especially in situations where such chemicals are not accessible to the farmers. In an experiment to demonstrate the killing potency of garlic, a 1 % garlic powder in water killed 100 % *Bacillus anthracis* (1.0 × 10^7^ cfu/ml) after three hours of exposure [40]. Eucalyptus leaf oil has bacteriostatic effect against *B. anthracis* [40]. There are claims that *Dicerocaryum eriocarpum* soaked in water can be used to disinfect the hands of individuals who touched the carcass of an animal that died from anthrax [41]. Hence, we propose that *Maesa lanceolata* and *Hypericum roeperianum* that had high total activities can be investigated as decontaminants or disinfectants, and active principles responsible for the mode of action should be isolated and characterized. *Maesa lanceolata* extracts had a high cytotoxicity and should not be used to protect animals against anthrax. Hypericum roeperianum extracts would be a good choice to use on animals due to its high total activity and high selectivity index.

The crude acetone extracts of the tested plant species revealed promising antibacterial activity against *B. anthracis.* Investigating the potential of these plant extracts to protect humans or animals against anthrax may lead to the development of new therapeutic agents. Since there are very few reports of *in vivo* studies of plant extracts against anthrax, such tests could be carried out using laboratory animal models. It would also be very interesting to determine the mechanism of activity.

## Abbreviations

INT: *p*-iodonitrotetrazolium violet
LC_50_: Lethal concentration killing 50 % of the cells
MIC: Minimum Inhibitory Concentration
MTT: 3-(4,5-dimethylthiazol-2-yl)-2, 5-diphenyltetrazolium bromide
SD: Standard Deviation
SI: Selectivity Index
TA: Total Activity
